# An Atypical Presentation of a Patient With Neurosarcoidosis: A Case Report

**DOI:** 10.7759/cureus.68229

**Published:** 2024-08-30

**Authors:** Ryan Tam, Stephen Howard, Massiel Jimenez-Artiles, Meenal Sharma, Fady Makdisy

**Affiliations:** 1 Internal Medicine, Lake Erie College of Osteopathic Medicine, Rochester, USA; 2 Physical Medicine and Rehabilitation, Lake Erie College of Osteopathic Medicine, Rochester, USA; 3 Internal Medicine, Rochester Regional Health, Rochester, USA; 4 Pathology and Laboratory Medicine, Rochester Regional Health, Rochester, USA

**Keywords:** leptomeningeal enhancement, granulomatous inflammation, sudden onset weakness, oculomotor nerve palsy, neurosarcoidosis

## Abstract

Neurosarcoidosis is a disease in which noncaseating granulomas, characteristic of sarcoidosis, are found within organs of the nervous system such as the brain and spinal cord. This case report highlights a 57-year-old male with worsening bilateral lower extremity weakness and numbness in addition to ptosis and oculomotor nerve palsy of the right eye. Computed tomography (CT) imaging showed mediastinal and hilar lymphadenopathy, which raised suspicion for neurosarcoidosis. Multiple biopsies were taken from lymph nodes in the mediastinal region, which resulted in non-necrotizing epithelioid cell granulomas, consistent with the suspected neurosarcoidosis. Medical providers must include neurosarcoidosis within a much broader differential diagnosis when encountering patients that present with a similar presentation shown in this case report so that treatment can be promptly initiated as soon as possible.

## Introduction

Sarcoidosis is a multisystem disease in which multiple noncaseating granulomas are present within various organs without a known trigger [1]. It most commonly affects the lungs and the lymphatic system [2]. The pathogenesis of sarcoidosis is not yet fully understood; however, there may be a genetic and environmental component to the disease [2]. Sarcoidosis that affects the nervous system is uncommon and occurs in approximately 5% to 10% of patients with sarcoidosis [2]. The prevalence of sarcoidosis in the United States ranges from 35 to 80 per 100000 people in those of African American descent and three to 10 per 100000 amongst Caucasians, with an average age of onset from 20 to 40 years old [3]. 

The diagnosis of neurosarcoidosis is a diagnosis of exclusion [4]. The differential diagnosis of neurosarcoidosis should be considered in a patient with sarcoidosis who develops neurological symptoms, however, there is no diagnostic marker [4]. Neurodiagnostic tests such as contrast-enhanced magnetic resonance imaging (MRI) are the imaging of choice, as this can be used to rule out other differential diagnoses such as infection or malignancy [4]. Findings on contrast-enhanced MRI typically include lesions within the hypothalamus, pituitary stalk, and other tissues within the central nervous system [4]. To diagnose neurosarcoidosis definitively, a biopsy must be done on an organ in the nervous system showing granulomatous inflammation [5]. F-18 fluorodeoxyglucose positron emission tomography and computed tomography (CT) have grown in popularity in testing patients with systemic sarcoidosis, as these imaging modalities help provide the most optimal biopsy site [4]. Sites that are biopsied with the lowest risk of complications include the skin, lymph nodes, and lungs [4]. However, if a diagnosis of neurosarcoidosis is suspected, a biopsy is most commonly taken from the meninges, brain, or spinal cord [4].

Treatment for neurosarcoidosis typically includes glucocorticoids, due to the inflammatory nature of the disease [4]. Most patients will receive prednisone for two to four weeks, although, for patients who are deteriorating, intravenous methylprednisolone may be given [4]. Those patients who fail a trial of corticosteroids may have the option to undergo immunosuppressant therapy or low-dose radiation [4]. For functional improvement, it has been shown that patients with neurosarcoidosis can experience rapid improvement with a comprehensive inpatient rehabilitation plan, which includes physical, occupational, and speech therapy for approximately two months to improve daily function [6].

## Case presentation

A 57-year-old African-American male with a past medical history significant for gastroesophageal reflux disease, benign prostatic hyperplasia, and lumbosacral radiculopathy presented to the emergency department with worsening bilateral lower extremity weakness and numbness. 

The patient stated that he felt fatigued two days before presenting to the emergency department, so he decided to sleep most of the day. Overnight, he woke up to use the bathroom but felt so weak that he was unable to ambulate back to his bed due to severe bilateral lower extremity weakness, which was new to the patient. As a result, he had to crawl on the ground until he got back to his bed. When the patient woke up in the morning, he noticed that he was unable to open his right eye and had continued to feel extremely lethargic and worsening bilateral lower extremity weakness; therefore, he decided to call for emergency medical services to be transported to the hospital. On further questioning, he denies any recent travels, sick contact exposures, or changes in lifestyle habits. The patient stated that his last known normal was the night before presentation with associated symptoms of fatigue and a six-month history of right ankle tenderness and swelling. 

**Figure 1:**
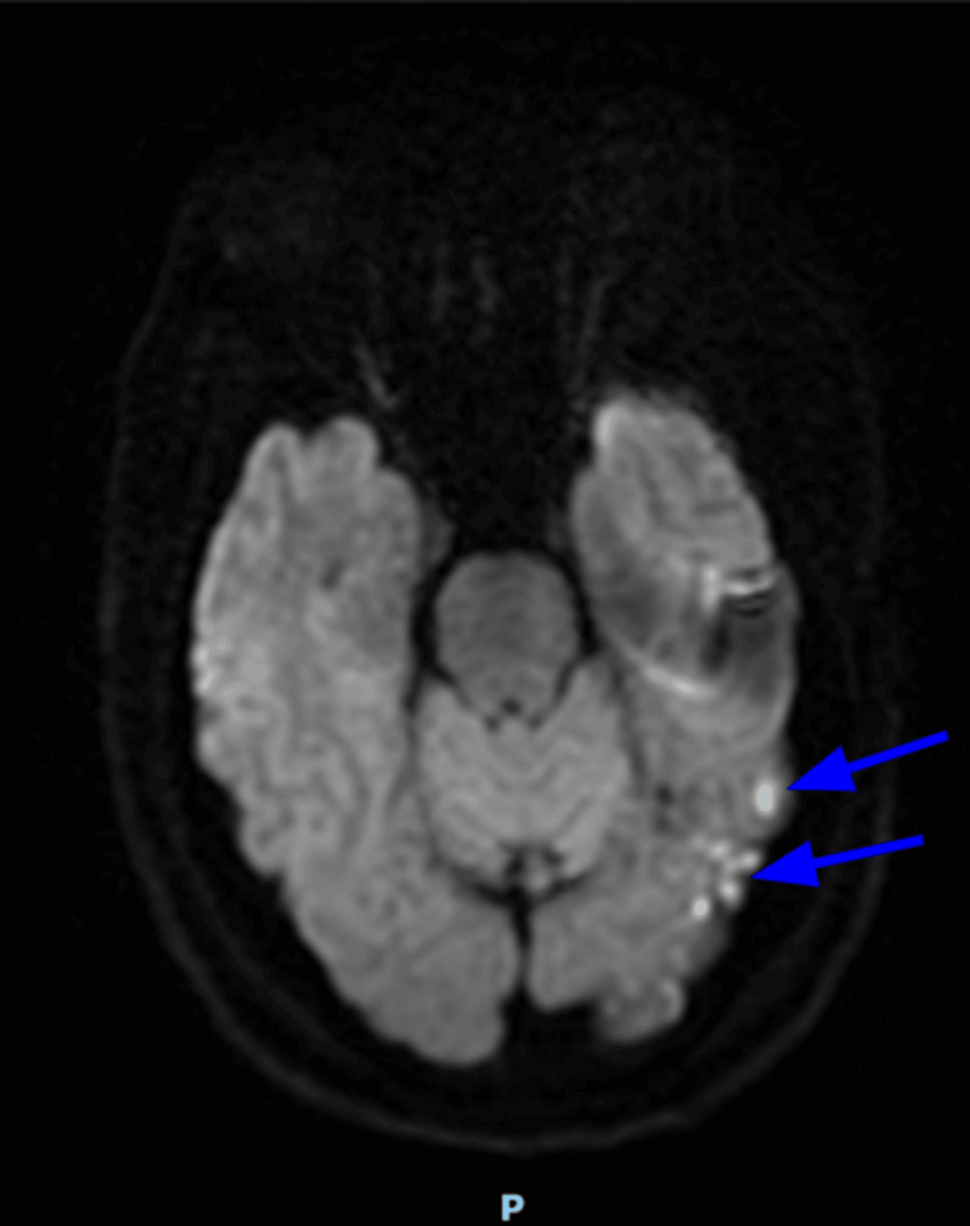
Axial MRI diffusion-weighted image with small foci of enhancement (blue arrows) along the posterior left temporal and left occipital lobes with leptomeningeal enhancement MRI: magnetic resonance imaging

**Figure 2:**
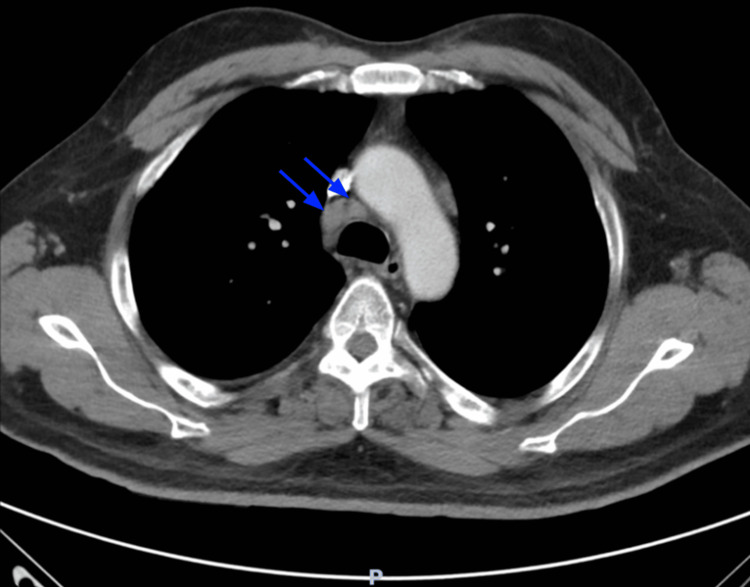
Axial CT chest with intravenous contrast showing 1.1 cm right paratracheal lymph nodes present (blue arrows) CT: computed tomography

During the current hospitalization course, physical examination was remarkable for the right eye ptosis with the right pupil being asymmetrically dilated and fixed when compared to the left pupil. Additionally, the patient had difficulty in voiding; therefore, a Foley catheter was inserted and was draining urine that was red and pinkish in color. Mild swelling and tenderness in the right ankle by the medial aspect and 4/5 strength on the right lower extremities with diminished sensation over the bilateral lower extremities were noted. Given the suspicious presentation of the patient with neurological symptoms that were concerning for an acute stroke, CT imaging of the head without contrast and an angiogram of the head and neck with contrast were ordered, which came back unremarkable. On further workup, an MRI of the brain was ordered to delineate if there is any minor stroke-like involvement (ischemia, subarachnoid hemorrhage, encephalitis, etc.) that may be missed with the negative CT imaging of the head in this patient with unspecific neurological symptoms. The MRI of the brain demonstrated small foci of enhancement along the posterior left temporal and left occipital lobes with an associated leptomeningeal enhancement that was consistent with sarcoidosis (Figure 1). This is further supported by CT imaging of the chest with contrast that demonstrated mediastinal and hilar lymphadenopathy (Figure 2).

**Figure 3:**
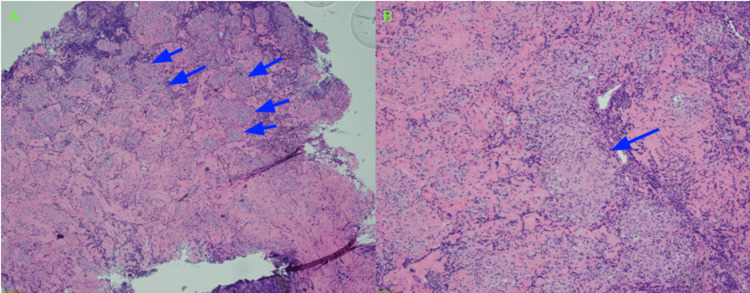
Hematoxylin and Eosin stained section of mediastinal lymph nodes with (A) multiple ill-formed non-necrotizing granulomas in a fibrotic lymph node (blue arrows) and (B) higher power view of the epithelioid cell granulomas (blue arrow)

Due to the high suspicion that the patient has neurosarcoidosis, neurology, cardiothoracic surgery, neurosurgery, and physiatry specialists were involved in the care of this patient. Finally, a decision was made to have the cervical mediastinoscopy procedure performed where multiple biopsies of lymph nodes in the mediastinal region were taken. The procedure was undertaken due to the location of the lymph nodes, which was believed to be easy to access as compared to the other locations that are more invasive with an increased complication rate. The biopsies were later evaluated by the pathology team, which showed scattered ill-formed non-necrotizing epithelioid cell granulomas, consistent with the suspected sarcoidosis diagnosis (Figure 3). Additionally, cerebrospinal fluid (CSF) analysis also showed an elevated angiotensin-converting enzyme at 5.2 units/liter (reference range: 0 to 2.5 units/liter), thereby further confirming the diagnosis of neurosarcoidosis.

## Discussion

Sarcoidosis is a systemic, inflammatory condition characterized by the formation of noncaseating granulomas classically involving the lungs. Any organ can be affected by sarcoidosis but involvement of the nervous system, neurosarcoidosis, is reported in only 5%-10% of cases. Cranial neuropathy is the most common manifestation within the subset of neurosarcoidosis, with the facial nerve most commonly affected [7]. This is due to granulomatous inflammation of the leptomeninges extending to the brain and spinal cord via the Virchow-Robin spaces that allow the communication between cervical lymphatic nodes and the brain [7]. On the other hand, the involvement of cranial nerves III, IV, V, VI, and VIII is less commonly affected [2]. With that being said, our case is unique given the combination of the acute onset of bilateral leg weakness with simultaneous urinary voiding difficulties in a patient with ptosis and an asymmetrically fixed and dilated pupil in his right eye displaying cranial nerve III palsy.

The 2018 Neurosarcoidosis Consortium Consensus was released as a guideline with several criteria that can help quantify whether the patient has a “definite,” “probable,” or “possible” diagnosis of neurosarcoidosis. To meet the “definite” diagnosis of neurosarcoidosis, there are two specific criteria to be met: 1) clinical presentation and diagnosis evaluation suggesting neurosarcoidosis by typical findings on MRI, CSF analysis, electromyography, and/or nerve conduction study after exclusion of other possible causes and 2) consistent nervous system pathology whether it is extraneural or isolated central nervous system sarcoidosis [3]. In the case of our patient, he meets the definite category based on his positive imaging findings of leptomeningeal enhancement in addition to the non-necrotizing granulomas noted on biopsy and positive CSF analysis. Although it would be preferable to also obtain neural tissue for histological analysis, this has been a major challenge as it can be associated with a high risk of morbidity and mortality [3]. 

After the diagnosis of neurosarcoidosis is made, it is important that treatment is promptly undertaken for both symptom management and disease remission. Glucocorticoids are commonly used as the first-line therapy for neurosarcoidosis, which has been demonstrated to have a desirable effect; however, there is a long tapering course and toxicity associated with its use [3]. Additionally, steroid-sparing agents, such as azathioprine, methotrexate, and mycophenolate mofetil, can be used but it takes several months to reach its full event [3]. Given the release of proinflammatory cytokines such as tumor necrosis factor-alpha (TNF-alpha) in the pathogenesis of inflammation within the nervous system, TNF-alpha inhibitors such as infliximab can be used, which have shown to be extremely efficacious [7]. In the treatment of our patient, high-dose intravenous methylprednisolone at 500 mg was started for five days followed by 1 mg/kg of oral prednisone. There were immediate improvements noted in the patient as he was able to demonstrate greater gradual recovery of his motor strength, sensory functions, and the resolution of the oculomotor nerve palsy of the right eye. Additionally, physical therapy, occupational therapy, and physiatry services were consulted to help the patient recover greater strength and function. The noted improvement in this patient’s physical status, with resolution of ptosis, pupillary reactivity to light and accommodation, and recovery of strength and sensation, demonstrated how comprehensive rehabilitation therapy may help reduce complications with steroid intake and improve the patient’s activities of daily living and quality of life [8].

## Conclusions

Although neurologic involvement is less common in patients with sarcoidosis, the symptoms can be extremely debilitating as seen in the case of our patient. In the case of this patient, the novelty exists in the combination of bilateral leg weakness in the setting of oculomotor nerve palsy and urinary retention, which was promptly treated with a tapering dose of steroids, with the desired effect. Thus, an early diagnosis will significantly benefit patients with this condition so that specific treatment can be directed with an interdisciplinary team approach to assist patients on their way to making meaningful functional recovery and improvement.
